# Reperfusion therapies for ischemic stroke in dementia and cognitive impairment: A systematic review and meta-analysis

**DOI:** 10.1177/17474930231220186

**Published:** 2023-12-27

**Authors:** Fouzi Bala, William Betzner, Benjamin Beland, Jennifer S McDonald, Aravind Ganesh

**Affiliations:** 1 Diagnostic and Interventional Neuroradiology Department, University Hospital of Tours, France; 2 Departments of Clinical Neurosciences and Community Health Sciences, Hotchkiss Brain Institute, University of Calgary Cumming School of Medicine, Calgary, AB, Canada; 3 Department of Radiology, Mayo Clinic College of Medicine, Mayo Clinic, Rochester, MN, USA; 4 Matheson Centre for Mental Health Research and Education, O’Brien Institute for Public Health, Calgary, AB, Canada

**Keywords:** Reperfusion, stroke, dementia, cognitive impairment, outcomes

## Abstract

**Background:**

Endovascular thrombectomy (EVT) and intravenous thrombolysis (IVT) have an unclear benefit in those with pre-stroke dementia or cognitive impairment, as these patients were often excluded from landmark stroke trials. We performed a systematic review and meta-analysis to assess the outcomes of IVT and EVT in these populations.

**Aims:**

Our systematic review, conforming to the Meta-Analysis of Observational Studies in Epidemiology guidelines, investigated studies on acute ischemic stroke patients with pre-stroke dementia or cognitive impairment treated with IVT or EVT. Primary outcome was favorable 90-day outcome (mRS 0–2). Secondary outcomes included 90-day mortality, symptomatic intracranial hemorrhage (SICH), and radiographic intracranial hemorrhage (ICH).

**Summary of review:**

Nine articles were identified, with five observational studies of IVT use in patients with (n = 1078) and without dementia (n = 2805) being selected for the meta-analysis. There were no significant differences in favorable outcome (adjusted OR: 0.61, 95% CI 0.24–1.59), mortality (unadjusted OR: 1.19, 95% CI 0.86–1.64), ICH (unadjusted OR: 1.32, 95% CI 0.79–2.19), and symptomatic ICH (unadjusted OR: 0.94, 95% CI 0.70–1.25) for patients undergoing IVT with pre-stroke dementia versus those without. One EVT study (n = 615 with dementia vs n = 9600 without) found no significant differences in outcomes apart from an increased odds of ICH for those with pre-existing dementia (adjusted OR: 1.57, 95% CI 1.03–2.40). A pooled analysis of three IVT studies showed no significant association of cognitive impairment (n = 93 vs n = 211 without) with all assessed outcomes, whereas a study of EVT found that pre-stroke cognitive impairment was associated with poor 90-day outcomes (mRS 3–6).

**Conclusion:**

These results suggest no substantial safety issues in the use of IVT or EVT for patients with pre-existing dementia or cognitive impairment compared to those without. However, the efficacy of these therapies in this demographic remains uncertain. Further rigorous studies that include a more nuanced outcome measurement approach are warranted.

**Registration:**

URL: https://www.crd.york.ac.uk/PROSPERO/; Unique identifier: CRD42021240499.

## Introduction

Patients with dementia have demonstrable evidence of cognitive impairment that represents a decline from their baseline and interferes with their ability to complete activities of daily living.^1^ Pre-stroke dementia is not uncommon with its prevalence estimated to be 12% in an analysis of the population-based Oxford Vascular Study.^2^ Whereas reperfusion therapies such as endovascular thrombectomy (EVT) and intravenous thrombolysis (IVT) have become the mainstay for the treatment of acute ischemic stroke for eligible patients,^3–5^ the benefit of these therapies in patients with pre-stroke dementia has not been well established.^4,6^

There is a lack of evidence from randomized-controlled trials for the use of reperfusion therapies in patients with pre-stroke dementia, partly because studies investigating reperfusion therapies have generally excluded patients with a pre-stroke modified Rankin Scale (mRS) score ⩾ 2.^4,5 [Bibr bibr7-17474930231220186]^ Further, the mRS was not initially designed for pre-stroke functional assessments.^7^ This essentially translates into an exclusion of patients with dementia who by definition have impairments in daily activities. Indeed, even patients in milder stages of dementia who remain independent for basic daily activities, yet have given up certain instrumental activities such as driving or work would fall into an mRS of 2, marking a transition from mild cognitive impairment (MCI) to dementia. Importantly, whereas cognitive impairment or dementia are not explicit contraindications for IVT or EVT, guidelines recommend careful considerations of relevant factors, such as goals of care, place of living, quality of life, and patient and family preferences before offering such therapies to these patients.^3,8,9^

Nevertheless, it seems that patients with dementia are typically excluded from reperfusion therapies in routine practice.^6^ The UNMASK-EVT study surveyed 607 stroke experts from around the world and determined factors that pushed participants toward or away from recommended reperfusion therapies (IVT or EVT) in hypothetical clinical scenarios. Surprisingly, the study found that MCI was the only comorbidity that showed consistently lower odds for offering EVT and thrombolysis in the adjusted model; this despite the fact that patients with MCI are by definition independent for instrumental and daily activities, unlike those with dementia.^6^ These results may be attributed to persisting concerns among stroke physicians about the increased risk of bleeding with reperfusion therapy in patients with dementia given the presumably high prevalence of white matter lesions and cerebral microbleeds in these patients.^10–12^

Given the paucity of high-quality evidence for the use of reperfusion therapies in patients with pre-stroke cognitive impairment and dementia, we sought to estimate the efficacy and safety of reperfusion therapies for acute ischemic stroke in this population using a systematic review and meta-analysis of observational studies.

## Methods

This meta-analysis is compliant with the Preferred Reporting Items for Systematic Reviews and Meta-Analyses (PRISMA) guidelines^13^ and was written according to the Meta-analysis of Observational Studies in Epidemiology (MOOSE) guidelines (checklist attached in Supplemental material).^14^

### Protocol and registration

The study was registered at PROSPERO, number CRD42021240499.

### Search strategy, inclusion, and exclusion criteria

We searched Ovid-Embase and MEDLINE for studies reporting outcomes in acute stroke patients with pre-stroke dementia or cognitive impairment treated with reperfusion therapy (IVT or EVT) from inception to 31 January 2023. No language or date restricitons were applied. Inclusion criteria were as follows: (1) randomized or observational studies of adult patients with cognitive impairment or dementia; (2) treated with IVT or EVT for acute ischemic stroke; and (3) including a minimum of 10 patients. Exclusion criteria included (1) case reports or series without any comparator groups (either untreated patients or patients without dementia or cognitive impairment) and (2) in vitro/animal studies. We searched the two databases using a combination of the following terms: (tissue plasminogen activator OR alteplase OR intravenous thrombolysis OR mechanical thrombectomy OR thrombectomy OR percutaneous thrombectomy OR endovascular therapy) AND (dementia OR cognitive impairment OR cognition) AND (stroke OR brain ischemia OR acute stroke OR arterial occlusion). See Supplemental material for the detailed search algorithm and the list of search terms that were employed in MEDLINE and Ovid Embase. Reference lists of included articles were also screened to identify additional studies that may have been missed during the initial search.

The titles and abstracts were screened by two authors (F.B. and B.B) using Covidence systematic review management software, and full texts of eligible studies were retrieved for inclusion. Conflicts were resolved by a third reviewer (A.G.). Qualifications of reviewers are included in the Supplemental material.

The data supporting this meta-analysis are available from the corresponding author on reasonable request.

### Data extraction

Two authors performed the data extraction which was then cross-checked by a third author. The following data were collected: study design, sample size, type of cognitive impairment, crude data, and unadjusted or adjusted odds ratios (ORs) with their 95% confidence interval (CI) for outcomes of interest. We contacted corresponding authors of included studies to request unpublished additional data and responses were received from one author.^15^

### Outcomes

The primary outcome was favorable outcome defined as mRS 0–2 at 90 days (or discharge if 90-day data were not available) in patients with versus without pre-stroke cognitive impairment or dementia. Although we were mainly interested in the outcome of return to baseline function, since patients with dementia have (by definition) some degree of dependency before stroke, no included study reported this outcome. Secondary outcomes included mortality at 90 days, symptomatic intracranial hemorrhage (SICH, according to definition used in included studies), and radiographic intracranial hemorrhage (ICH).

### Statistical analyses

ORs and their corresponding 95% CIs were calculated for all the outcomes using random-effects models (Der Simonian and Laird method). In addition, we pooled adjusted effect size estimates and the associated 95% CIs for studies that provided adjusted ORs, using the admetan command in STATA. As we did not have the individual patient data for these studies, we recorded the variables that were used for adjustment in each of these studies, as presented in the “Results” section.

Heterogeneity was assessed using the chi-square test and p ⩽ 0.10 was considered heterogeneous. Statistical tests were two-sided and p < 0.05 was considered statistically significant. Analyses were performed with the STATA Statistical Software Release 17 for Windows (StataCorp LP).

### Risk of bias in included studies

Two reviewers independently assessed each study using the Quality In Prognosis Studies (QUIPS) tool,^16^ and disagreement was resolved by a third reviewer. Risk of bias for each domain was rated as low, moderate, or high following detailed instructions. Risk of publication bias was assessed using funnel plots.

Test for funnel plot asymmetry (Egger’s linear regression test) for a given outcome was performed if at least 10 studies were available for analysis.^17^

## Results

### Included studies

**Figure 1.**
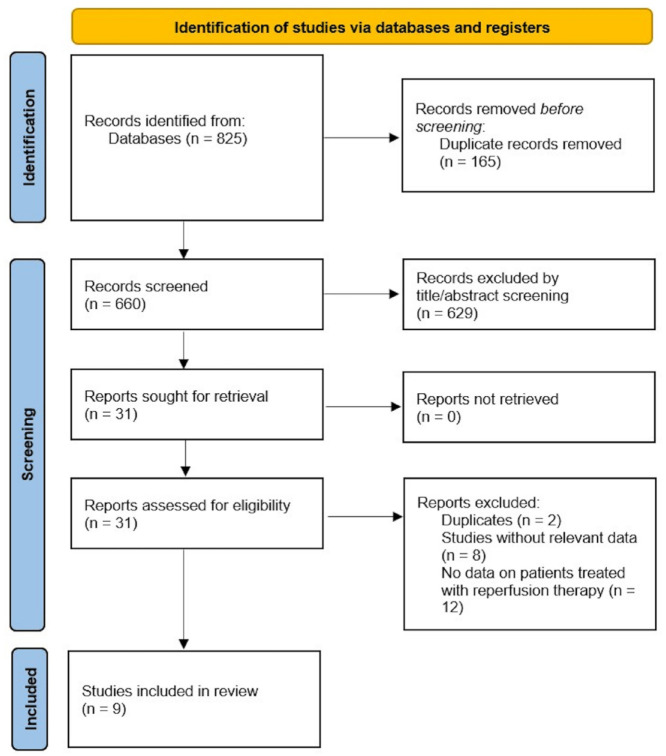
PRISMA flow diagram for study selection.

The initial search resulted in 825 articles. After removal of duplicates and title and abstract screening, 31 articles remained for full-text review (Figure 1).

We included nine articles in total (Table 1): five articles of patients with versus without pre-stroke dementia treated with IVT,^15,18–21^ and one article in patients with versus without pre-stroke dementia treated with EVT.^22^ For pre-stroke cognitive impairment, two articles were in patients treated with IVT^23,24^ and one with EVT.^25^ One study included only patients ⩾ 80 years old.^19^ The meta-analysis was performed in the five articles of patients with dementia versus without dementia treated with IVT.

**Table 1.** Summary data of the included studies.

Author	Study groups	N	Age (mean)	% male	NIHSS (mean)	90-day mRS 0–2, n (%)	90-day mortality, n (%)	SICH, n (%)	ICH, n (%)
Studies comparing patients treated with IVT with and without pre-stroke dementia									
Alshekhlee et al.^18^	Dementia	207	81	35	–	–	36 (17)	–	12 (6)
	No dementia	621	81	34	–	–	90 (14)	–	28 (4)
Saposnik et al.^20 a^	Dementia	99	81	36	6	10 (10)	27 (27)	11 (11)	19 (19)
	No dementia	99	83	34	3	17 (17)	28 (28)	11 (11)	15 (15)
Busl et al.^19^	Dementia	21	86	24	16	7 (33)	13 (62)	3 (14)	–
	No dementia	132	86	32	15	76 (57)	41 (31)	7 (5)	–
Nasr et al.^15^	Dementia	657	82	34	–	–	84 (13)	52 (8)	–
	No dementia	1314	83	35	–	–	178 (14)	120 (9)	–
Zupanic et al.^21^	Dementia	94	83	42	12	4 (5)	18 (22)	7 (7)	–
	No dementia	639	81	45	10	189 (33)	107 (19)	46 (6)	–
Studies comparing patients treated with EVT with and without pre-stroke dementia									
Saber et al.^22 b^	Dementia	615	–	–	–	–	131 (21)	–	185 (30)
	No dementia	9600	–	–	–	–	1075 (11)	–	2035 (21)
Studies comparing patients treated with IVT with and without pre-stroke cognitive impairment									
Murao et al.^23^	PCI	31	73	52	11	19 (61)	2 (7)	0 (0)	–
	No PCI	68	64	53	14	44 (65)	6 (9)	3 (4)	–
Murao et al.^24^	PCI	62	77	42	9	35 (56)	3 (5)	7 (11)	–
	No PCI	143	67	56	8	102 (71)	7 (5)	5 (3)	–
Studies comparing patients treated with EVT with and without pre-stroke cognitive impairment									
Kanamaru et al.^25^	PCI	23	79	52	15	5 (22)	2 (9)	1 (4)	4 (17)
	No PCI	90	72	78	16	47 (52)	4 (4)	8 (9)	22 (24)

EVT, endovascular thrombectomy; ICH, intracranial hemorrhage; IVT, intravenous thrombolysis; mRS, modified Rankin Scale; NIHSS: National Institutes of Health Stroke Scale; PCI, pre-stroke cognitive impairment; SICH, symptomatic intracranial hemorrhage.

a Data are from the matched groups.

b Mortality data were available only at discharge.

None of the studies systematically reported dementia severity nor did they stratify results based on severity. Three of the studies that reported cognitive impairment reported cognitive impairment in terms of an IQCODE score, although their definition varied and outcomes were not stratified by severity. One study defined pre-existing cognitive impairment (no dementia) as a score greater than 3 but less than 3.44,^23^ while the other described mild-to-moderate cognitive impairment as score greater than 3^24^ and the final defining it as a score greater than 3.4.^25^ Several studies justified omitting dementia and cognitive impairment severity based on pragmatic reasons, such as difficulties ascertaining baseline function and dementia severity in an acute stroke setting^19–21^ or the use of the National Inpatient Sample (NIS) which does not include baseline functional status^18,22^ As such, results are reported in a binary fashion, based on the presence or absence of pre-existing dementia or cognitive impairment.

### Studies in patients with and without dementia treated with IVT

Five studies compared outcomes between patients with (n = 1078) and without dementia (n = 2805) treated with intravenous thrombolysis.^15,18–21^ All studies were observational retrospective cohorts and multi-center except for one which was single-center.^19^ Variables used for adjustment varied between studies and are shown in Supplemental material (Table 1).

**Figure 2.**
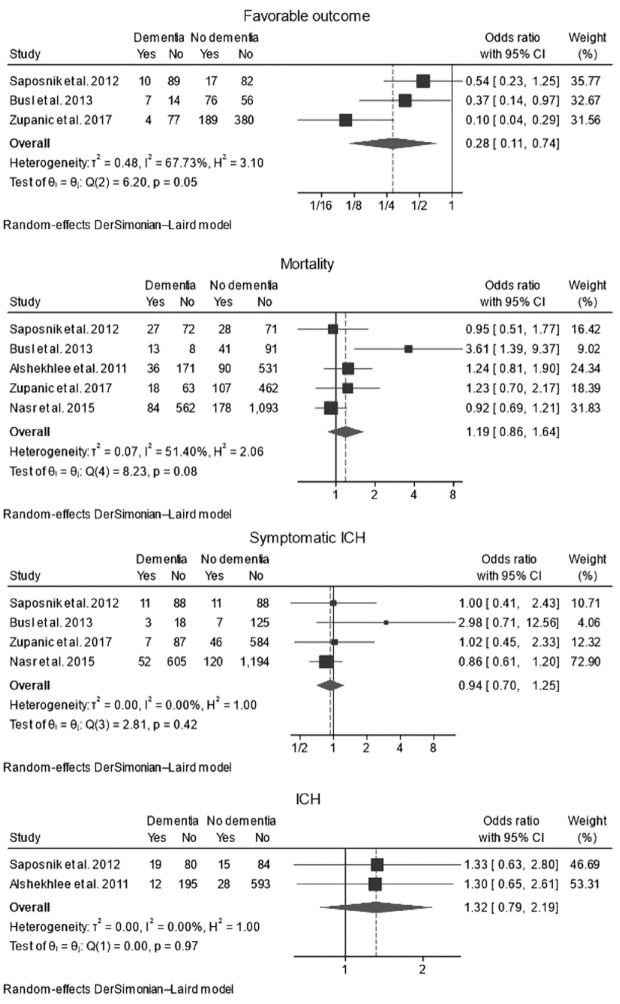
Forest plots with random-effect model of pooled unadjusted OR in patients with and without pre-stroke dementia treated with intravenous thrombolysis. (a) mRS 0–2 at 90 days; (b) mortality at 90 days; (c) SICH; (d) ICH. mRS, modified Rankin Scale; CI, confidence interval; ICH, intracranial hemorrhage; SICH, symptomatic ICH.

Three studies reported data on favorable outcome using mRS 0–2 at 90 days^21^ or at discharge^20^ (Figure 2(a)). In one study, favorable outcome was defined as discharge to home or rehabilitation facility.^19^ The pooled rate of favorable outcome was 12.2% (95% CI 3.0–26.0%, I^2^ = 80.5%) in patients with dementia, and 35.2% (95% CI 17.8–55.0%, I^2^ = 95.5%) in patients without dementia. Dementia was associated with a lower likelihood of favorable outcome using the crude data (unadjusted OR: 0.28, 95% CI 0.11–0.74, p = 0.01, I^2^ = 67.7%); however, when the adjusted ORs were pooled from the included studies (n = 2), the difference in odds of favorable outcome was not significant anymore (adjusted OR: 0.61, 95% CI 0.24–1.59, p = 0.31, I^2^ = 69.6%) (Supplemental material (Figure 1A)).

Mortality data were available from all five studies (Figure 2(b)). There was no significant difference in the rate of mortality between patients with (23.9% (95% CI 15.0–34.1%, I^2^ = 88.6%)) and without dementia (19.8% (95% CI 15.0%–25.0%, I^2^ = 88.4%)), with an unadjusted OR of 1.19, 95% CI 0.86–1.64, p = 0.30, I^2^ = 51.4%. Results were similar when examining in the adjusted pooled ORs (Supplemental material (Figure 1B)).

Rates of symptomatic ICH were reported in four studies (Figure 2(c)) and were similar between both groups: 7.8% (95% CI 6.0–9.8%, I^2^ = 0%) versus 8.1% (95% CI 6.5–9.9%, I^2^ = 33.3%) in patients with and without dementia, respectively, with an unadjusted OR of 0.94, 95% CI 0.70–1.25, p = 0.65, I^2^ = 0%.

Rates of any ICH (whether symptomatic or not) were reported in two studies (Figure 2(c)) and were not different between the two groups (unadjusted OR: 1.32, 95% CI 0.79–2.19, p = 0.29, I^2^ = 0%).

### Studies in patients with and without dementia treated with EVT

We found one study assessing outcomes of patients with dementia versus no dementia treated with EVT (Table 1).^22^ Saber et al. did not find a significant difference between in-hospital mortality, discharge disposition to home, and pre-stroke dementia in their cohort of 10,215 acute ischemic patients undertaken to EVT (6% with dementia) after adjustment for stroke severity. However, dementia was associated with increased odds of radiographic ICH (adjusted OR: 1.57 95% CI 1.03–2.40).^22^

### Studies in patients with and without cognitive impairment treated with IVT

We found two articles from one group on outcomes of patients with cognitive impairment treated with IVT.^23,24^ One article was from an observational prospective multi-center study which evaluated the effect of the pre-existing cognitive impairment on the outcome in patients treated by thrombolytic therapy for acute ischemic stroke and included 205 patients^24^ and another article from two biomarker studies Biostroke and Strokdem which included 99 patients.^23^ The pooled analysis of these three studies did not show a significant association of cognitive impairment with all assessed outcomes (mRS 0–1, 0–2, death at 90 days, and symptomatic ICH).^24^

### Studies in patients with and without cognitive impairment treated with EVT

A study by Kanamaru et al.^25^ looked up the association between pre-stroke cognitive impairment and functional outcomes in patients treated with EVT. They included 113 patients between 2016 and 2018 from one center in Japan and found that pre-stroke cognitive impairment was associated with poor outcomes (mRS 3–6) at 90 days.^25^ However, symptomatic and radiographic ICH rates were not different between both groups. It should be noted that there were only 23 patients in total with pre-stroke cognitive impairment in this study.

### Quality of included studies

Using the QUIPS tool, five of the nine included studies were found to have a low risk of bias, three had a moderate risk, and one had a high risk (Supplemental material (Figure 2)). A funnel plot assessment was not completed as there were fewer than 10 included studies per outcome of interest.

## Discussion

In this systematic review and meta-analysis of observational studies examining the safety and efficacy of IVT and EVT reperfusion therapies in patients with pre-existing dementia or cognitive impairment who experienced an acute ischemic stroke, we found no significant differences in outcomes including ICH, symptomatic ICH, or mortality for patients with pre-stroke dementia or cognitive impairment undergoing these therapies. In addition, although the crude data showed a lower likelihood of favorable outcome (mRS 0–2) after IVT in those with dementia versus those without, the pooled adjusted ORs from two of the included studies showed no significant differences.

The absence of significant differences in ICH, symptomatic ICH, and mortality between treated patients with versus without dementia can help reassure physicians about the general safety of reperfusion therapies in patients with pre-morbid dementia. Furthermore, our finding of no significant difference in both unadjusted and adjusted mortality is particularly reassuring as this contrasts with our prior meta-analysis in patients with pre-morbid disability (another challenging patient population), in which we observed higher mortality rates in patients with versus without pre-morbid disability undergoing either EVT^26^ or IVT^27^ reperfusion therapies.

Nonetheless, the efficacy of IVT and EVT in patients with dementia remains an area of uncertainty. Of particular note, our systematic review aiming to capture randomized and observational studies of patients with pre-existing dementia or cognitive impairment only identified nine observational studies, five of which were included in the meta-analysis. Importantly, the current literature lacks robust comparative analyses between treated and untreated patients with dementia, making it challenging to comprehend the real-world treatment effects. For example, we found that more than 1 in 10 patients with dementia can have a favorable outcome after IVT; while this is a helpful proportion to help inform treatment expectations, knowing how this rate compares to those who do not receive IVT would be even more meaningful. While our analysis noted that patients with dementia had generally worse functional outcomes compared to those without dementia after treatment, this outcome is not surprising given their impaired pre-morbid state. As such, the assessment of “favorable outcome” in such patients needs to be reconsidered since most patients with dementia who by definition have some functional dependence on others cannot be reasonably expected to achieve an mRS of 0–2. Future research requires the use of more inclusive and appropriate definitions, such as a return to pre-morbid baseline or a change in mRS from pre-stroke to post-stroke (delta-mRS),^28^ and more robust study design to confirm the safety of IVT and EVT use for people with pre-existing dementia or cognitive impairment. Using these definitions can provide a more nuanced understanding of the real benefits these treatments might offer to patients with pre-existing cognitive impairments.

More robust study design also relates to the question of dementia and cognitive impairment severity in relation to reperfusion outcomes. Although it stands to reason that outcomes likely vary based on dementia stage, none of the studies explicitly reported this, nor did they stratify results based on severity. Pragmatic issues in ascertaining baseline functional status are often to blame; however, study design can impact this as well. For example, in the cognitive impairment studies, the IQCODE cut-off for cognitive impairment varied, and no results were shown stratifying IQCODE scores and outcomes.^23–25^ As such, reperfusion outcomes based on mild to moderate versus severe dementia remain unknown.

Our findings reinforce the recent scientific statement from the American Heart Association/American Stroke Association on the necessity of including patients with pre-stroke disability or dementia in clinical trials studying reperfusion therapies.^29^ Given that approximately 12% of the population has pre-stroke dementia, these patients should not be overlooked in clinical trials and treatment plans.^2^ Excluding them may lead to a treatment bias and a lack of tailored strategies for caring for this population in an acute stroke setting.

In addition, we strongly urge that future research be guided by patients’ and caregivers’ inputs. Understanding their preferences, goals, and concerns may provide valuable insights into the treatment decision-making process and improve patient-centered care. Furthermore, understanding the reasons behind current physician practices is necessary. These questions are being explored, for instance, in the ongoing mixed-methods SEED (Stroke-related Experiences and priorities of Elderly living with Dementia, their family caregivers, and physicians) study.^30^ SEED aims to explore the perspectives surrounding acute stroke care of these key stakeholders to help inform future clinical trial design and personalized care.

In conclusion, our study highlights the need for further high-quality research to better understand the benefits and risks of reperfusion therapies in patients with pre-existing dementia or cognitive impairment. While our results demonstrate no substantial safety concerns, the efficacy of these therapies in this patient population remains uncertain and necessitates a more inclusive and nuanced approach to outcome measurement. Our meta-analysis found high I^2^ values, suggesting considerable heterogeneity between included studies. In addition, we were only able to perform a meta-analysis on studies pertaining to IVT use in those with dementia, and all these studies were retrospective with their inherent limitation of selection bias. However, to our knowledge, this is the first meta-analysis trying to pool the data of studies assessing stroke patients with cognitive impairment receiving reperfusion therapy. These limitations further underscore the need for more methodologically rigorous study designs for exploring the use of reperfusion therapies for people living with dementia. Such studies would pave the way for more inclusive, equitable, and effective stroke care.

## Supplemental Material

sj-docx-1-wso-10.1177_17474930231220186 – Supplemental material for Reperfusion therapies for ischemic stroke in dementia and cognitive impairment: A systematic review and meta-analysis Supplemental material, sj-docx-1-wso-10.1177_17474930231220186 for Reperfusion therapies for ischemic stroke in dementia and cognitive impairment: A systematic review and meta-analysis by Fouzi Bala, William Betzner, Benjamin Beland, Jennifer S McDonald and Aravind Ganesh in International Journal of Stroke
