# Supplementary material for: Reperfusion therapies for ischemic stroke in dementia and cognitive impairment: A systematic review and meta-analysis
Source: Int J Stroke. 2023 Dec 27;19(8):867–75. doi: 10.1177/17474930231220186 (PMC11409564; doi:10.1177/17474930231220186)
Supplement: sj-docx-1-wso-10.1177_17474930231220186 – Supplemental material for Reperfusion therapies for ischemic stroke in dementia and cognitive impairment: A systematic review and meta-analysis [file sj-docx-1-wso-10.1177_17474930231220186.docx]

**SUPPLEMENTAL MATERIAL**

**Reperfusion Therapies for Ischemic Stroke in Dementia and Cognitive Impairment: A Systematic Review and Meta-Analysis**

**Table of Contents:**

**2 – OVID Embase Search Strategy**

**3 – Reviewer Qualifications**

**4 – Supplemental Table 1**

**5 – Supplemental Figure 1**

**6 – Supplemental Figure 2**

**7-8 – Meta-analysis of Observational Studies in Epidemiology (MOOSE) Checklist**

**OVID Embase search strategy**

1. mechanical thrombectomy/

2. thrombectomy/

3. percutaneous thrombectomy/

4. (endovascular treatment or endovascular therapy or intraarterial revascularization or thrombolysis or tPA).tw.

5. blood clot lysis/

6. tissue plasminogen activator/

7. alteplase/

8. 1 or 2 or 3 or 4 or 5 or 6 or 7

9. cognitive decline/

10. Dementia/

11. cognitive impairment/

12. cognition/

13. 9 or 10 or 11 or 12

14. cerebrovascular accident/

15. brain ischemia/

16. (acute stroke or acute ischemic stroke or stroke or intracranial occlusion or arterial occlusion).tw.

17. 14 or 15 or 16

18. 8 and 13 and 17

**OVID Medline search strategy**

1. Thrombolytic Therapy/ or Thrombectomy/ or Mechanical Thrombolysis/

2. (endovascular and (therapy or treatment or therapeutics)).tw.

3. (intra-arterial and revascularization).tw.

4. (mechanical and thrombectomy).tw.

5. Thrombolysis / or tissue plasminogen activator / or Alteplase /

6. (thrombolysis treatment or thrombolysis therapy).tw.

7. (intravenous and thrombolysis).tw.

8. 1 or 2 or 3 or 4 or 5 or 6 or 7

9. cognitive decline/

10. Dementia/

11. cognitive impairment/

12. cognition/

13. 9 or 10 or 11 or 12

14. Stroke/

15. Brain Ischemia/ or Ischemia/

16. (acute stroke or acute ischemi* or stroke or intracranial occlusion or arterial occlusion).tw.

17. 14 or 15 or 16

18. 8 and 13 and 17

**Reviewers:**

Fouzi Bala MD, MSc: Neurointerventionalist

Benjamin Beland MD: Senior Neurology Resident-Physician

Aravind Ganesh MD DPhil(Oxon) FRCPC: Stroke and Cognitive Neurologist

**Supplement Table 1.** Summary of the variables other than pre-stroke dementia that were adjusted for in the available adjusted analyses for the included studies.

| **Study** | **Variables adjusted for in multivariable models** |
| --- | --- |
| Saposnik et al. 2012^20^ | Age, sex, NIHSS score, stroke subtype (lacunar vs. other), vascular risk factors, level of consciousness on arrival, dysphagia, glucose and creatinine levels on admission, arrival to hospital from a residence (home vs. other), pre-admission dependency, palliative care, stroke unit admission, thrombolysis, physiotherapy, and arrival by ambulance |
| Busl et al. 2013^19^ | Age, NIHSS score, symptomatic intracranial hemorrhage, thrombolysis, EVT |
| Zupanic et al. 2017^21^ | Age, sex, NIHSS score, living alone (not in a nursing home), pre-admission dependency, number of medications |

# EVT, endovascular thrombectomy; NIHSS, National Institutes of Health Stroke Scale; TIA, transient ischemic attack.

**Supplemental Figure 1**

| **** |
| --- |
| **** |

**Figure 1. Forest plots with random-effect model of pooled adjusted odds ratio.** (A) favorable outcome, (B) mortality.

Pooling of effect size estimates was not performed for symptomatic intracranial hemorrhage (SICH) and intracranial hemorrhage because of limited data: 1 study for SICH and no study for ICH.

| Study ID | Study Participation | Study Attrition | Prognostic Factor Measurement | Outcome Measurement | Study Confounding | Statistical Analysis and Reporting | Overall |
| --- | --- | --- | --- | --- | --- | --- | --- |
| Alshekhlee et al. 2011 | Low | Low | Moderate | Moderate | Moderate | Low | Moderate |
| Busl et al.  2013 | Low | Low | Moderate | Low | Low | Low | Low |
| Sapsonik et al. 2012 | Low | Moderate | Low | Low | Low | Low | Low |
| Zupanic et al. 2017 | Low | High | Moderate | Low | Moderate | Low | Moderate |
| Nasr et al.  2015 | Moderate | High | High | Moderate | High | High | High |
| Murao et al. 2013 | Low | Low | Low | Low | Low | Low | Low |
| Murao et al. 2014 | Low | Low | Low | Low | Low | Low | Low |
| Kanamaru et al. 2021 | Low | Low | Low | Low | Low | Low | Low |
| Saber et al. 2022 | Low | Low | Low | Moderate | Moderate | Low | Moderate |

**Figure 2: Risk of bias assessments for the studies of reperfusion therapies in patients with pre-existing dementia or cognitive impairment.**

**MOOSE Checklist for Meta-analyses of Observational Studies**

| **Item No** | **Recommendation** | **Reported on Page No** |
| --- | --- | --- |
| Reporting of background should include | | |
| 1 | Problem definition | 2 |
| 2 | Hypothesis statement | 2 |
| 3 | Description of study outcome(s) | 2 |
| 4 | Type of exposure or intervention used | 2 |
| 5 | Type of study designs used | 5 |
| 6 | Study population | 2 |
| Reporting of search strategy should include | | |
| 7 | Qualifications of searchers (eg, librarians and investigators) | Suppl. |
| 8 | Search strategy, including time period included in the synthesis and key words | 6 |
| 9 | Effort to include all available studies, including contact with authors | 7 |
| 10 | Databases and registries searched | 6 |
| 11 | Search software used, name and version, including special features used (eg, explosion) | 6 |
| 12 | Use of hand searching (eg, reference lists of obtained articles) | 6 |
| 13 | List of citations located and those excluded, including justification | 6 |
| 14 | Method of addressing articles published in languages other than English | 6 |
| 15 | Method of handling abstracts and unpublished studies | 6-7 |
| 16 | Description of any contact with authors | 7 |
| Reporting of methods should include | | |
| 17 | Description of relevance or appropriateness of studies assembled for assessing the hypothesis to be tested | 7 |
| 18 | Rationale for the selection and coding of data (eg, sound clinical principles or convenience) | 7-8 |
| 19 | Documentation of how data were classified and coded (eg, multiple raters, blinding and interrater reliability) | 6-7 |
| 20 | Assessment of confounding (eg, comparability of cases and controls in studies where appropriate) | 7 |
| 21 | Assessment of study quality, including blinding of quality assessors, stratification or regression on possible predictors of study results | 8 |
| 22 | Assessment of heterogeneity | 8 |
| 23 | Description of statistical methods (eg, complete description of fixed or random effects models, justification of whether the chosen models account for predictors of study results, dose-response models, or cumulative meta-analysis) in sufficient detail to be replicated | 7-8 |
| 24 | Provision of appropriate tables and graphics | 6 |
| Reporting of results should include | | |
| 25 | Graphic summarizing individual study estimates and overall estimate | Figure 2 + Suppl. |
| 26 | Table giving descriptive information for each study included | Table 1 |
| 27 | Results of sensitivity testing (eg, subgroup analysis) | 9-11 |
| 28 | Indication of statistical uncertainty of findings | 13-15 |

| **Item No** | **Recommendation** | **Reported on Page No** |
| --- | --- | --- |
| Reporting of discussion should include | | |
| 29 | Quantitative assessment of bias (eg, publication bias) | 12 |
| 30 | Justification for exclusion (eg, exclusion of non-English language citations) | Figure 1 |
| 31 | Assessment of quality of included studies | Suppl. |
| Reporting of conclusions should include | | |
| 32 | Consideration of alternative explanations for observed results | 13-14 |
| 33 | Generalization of the conclusions (ie, appropriate for the data presented and within the domain of the literature review) | 15 |
| 34 | Guidelines for future research | 14-15 |
| 35 | Disclosure of funding source | 16 |

*From*: Stroup DF, Berlin JA, Morton SC, et al, for the Meta-analysis Of Observational Studies in Epidemiology (MOOSE) Group. Meta-analysis of Observational Studies in Epidemiology. A Proposal for Reporting. *JAMA*. 2000;283(15):2008-2012. doi: 10.1001/jama.283.15.2008.
